# Clinical Salvage Approaches for Surgical Site Infection After Autologous Microtia Reconstruction

**DOI:** 10.3390/jcm15031064

**Published:** 2026-01-29

**Authors:** Kap Sung Oh, Wonseok Cho, Junekyu Kim, Kyu Nam Kim

**Affiliations:** Department of Plastic and Reconstructive Surgery, Kangbuk Samsung Hospital, Sungkyunkwan University School of Medicine, Seoul 03181, Republic of Korea; kapsung.oh@samsung.com (K.S.O.); wscws26@naver.com (W.C.); j7181.kim@samsung.com (J.K.)

**Keywords:** microtia, surgical wound infection, costal cartilage, negative-pressure wound therapy, reconstructive surgical procedures

## Abstract

**Background/Objectives**: Surgical site infection (SSI) after autologous rib cartilage microtia reconstruction is an uncommon but potentially devastating complication, as infection of the avascular cartilage framework can rapidly lead to partial or complete framework loss. Traditional management often favored aggressive debridement or framework removal, resulting in significant deformity. This study aimed to evaluate salvage-oriented management strategies and to propose a structured treatment algorithm for SSI following microtia reconstruction. **Methods**: A retrospective case series was conducted of patients who developed SSI after autologous rib cartilage microtia reconstruction between March 2021 and November 2025. SSI was defined by clinical and surveillance criteria requiring intervention beyond routine postoperative care. Nine patients were included. Management strategies were analyzed with respect to infection control, framework preservation, and wound healing outcomes. **Results**: SSI occurred at variable time points, ranging from early postoperative infection to delayed and late-onset presentations. Identified pathogens included Gram-positive cocci and multidrug-resistant Gram-negative organisms. Negative-pressure wound therapy (NPWT) was applied in all cases with wound dehiscence, persistent drainage, or cartilage exposure. Conservative staged debridement was performed only after clear demarcation of nonviable tissue. Overall auricular framework preservation was achieved in 100% of patients, with no cases requiring complete framework removal, although limited cartilage loss occurred in select cases. These outcomes demonstrate the clinical feasibility and effectiveness of salvage-oriented management across heterogeneous infection scenarios. **Conclusions**: SSI following autologous microtia reconstruction can be effectively salvaged without routine framework removal through a structured, timing-based algorithm emphasizing early culture-guided antimicrobial therapy, NPWT, and conservative staged intervention. This salvage-oriented approach provides a clinically relevant and reproducible framework for preserving auricular structure while minimizing morbidity, even in infections involving multidrug-resistant organisms.

## 1. Introduction

Microtia is a congenital deformity of the external ear characterized by the partial or complete absence of the auricle, often accompanied by external auditory canal atresia and functional hearing impairment [1,2]. Autologous rib cartilage reconstruction remains the gold-standard technique for microtia repair due to its long-term structural stability and natural esthetic outcomes [1,2,3]. Despite these advantages, this multi-staged procedure is uniquely vulnerable to postoperative complications, particularly surgical site infection (SSI) [4,5,6]. Because the rib cartilage framework is avascular and surrounded by a relatively limited soft-tissue envelope, infection can rapidly compromise both soft-tissue viability and structural integrity, placing the entire reconstructed framework at risk of partial or complete loss [5,6]. Loss of the reconstructed framework represents one of the most devastating complications for patients, frequently necessitating complex secondary reconstructions and imposing substantial functional, esthetic, and psychological burdens [7,8].

Recent epidemiologic data underscore the clinical significance of SSI in microtia surgery [4,9,10,11,12]. Reported rates of wound complications and infection after autologous microtia reconstruction range from approximately 2% to 5% in large retrospective series and database-based analyses [4,9,10,11,12]. Large-scale analyses have demonstrated that Gram-positive organisms, particularly *Staphylococcus aureus* and coagulase-negative *Staphylococci*, remain the predominant pathogens [10,12]. However, Gram-negative organisms—including *Enterobacter* species and *Pseudomonas aeruginosa*—constitute a substantial proportion of infections and are often associated with multidrug resistance [10,13]. This microbiologic heterogeneity highlights the importance of early pathogen identification and a timely transition to targeted antimicrobial therapy to prevent progressive soft-tissue breakdown and cartilage necrosis [4,9,10].

The management of SSI in microtia reconstruction is further complicated by heterogeneity in timing and local tissue conditions. Infections may occur during the early postoperative period following rib cartilage framework implantation or ear elevation, while delayed infections can arise months or even decades later, sometimes precipitated by minor trauma [14,15]. The quality of the soft-tissue envelope, prior flap or graft history, and patient-specific factors collectively influence the feasibility of framework preservation [16,17].

Historically, aggressive debridement—including the complete removal of the cartilage framework—was frequently advocated once infection threatened cartilage viability [18,19]. Although effective for infection control, such radical approaches often resulted in severe auricular deformity and necessitated technically demanding secondary reconstructions [5,14,15]. Over the past decade, advances in antimicrobial strategies, negative-pressure wound therapy (NPWT), and conservative staged debridement have shifted clinical practice toward salvage-oriented management, emphasizing framework preservation rather than immediate removal [5,20,21,22]. In particular, NPWT has been shown to stabilize the wound environment by promoting continuous drainage and granulation tissue formation over exposed cartilage in selected cases of microtia reconstruction [5,20,21,22].

Nevertheless, the existing literature on salvage management remains fragmented. Most reports focus on isolated techniques or small case series and fail to integrate infection control, debridement strategy, and definitive soft-tissue coverage into a unified management pathway [5,21,22]. Prior studies also emphasize that the effectiveness of NPWT depends on wound size, soft-tissue availability, and early clinical response, underscoring the need for timing-based, algorithmic decision-making rather than prolonged reliance on a single modality [5,21,22]. Despite the recognized importance of framework preservation, clear and standardized guidelines for comprehensive SSI management following microtia reconstruction remain lacking.

Accordingly, this study synthesizes real-world clinical experience from patients who developed SSI after autologous microtia reconstruction. Rather than focusing on infection incidence, this study examines clinical decision-making and outcomes within the context of a stepwise salvage algorithm by analyzing shared clinical patterns, pathogen profiles, wound characteristics, and responses to staged interventions. These insights are intended to support surgeons in preserving the reconstructed framework while minimizing morbidity and avoiding unnecessary framework removal.

## 2. Materials and Methods

### 2.1. Study Design and Patient Selection

This retrospective case series included patients who developed surgical site infection (SSI) following autologous rib cartilage-based microtia reconstruction and were managed at our institution between March 2021 and November 2025. Autologous rib cartilage-based microtia reconstruction consisted of initial rib cartilage framework grafting, auricular elevation, and secondary touch-up procedures [18,19]. Hereafter, this procedure is referred to as autologous microtia reconstruction.

SSI was defined according to established clinical and surveillance criteria, including wound dehiscence, purulent discharge, localized erythema or tenderness, and/or exposure of the auricular framework requiring medical or surgical intervention beyond routine postoperative care [23,24,25]. These criteria are consistent with internationally accepted definitions of superficial and deep incisional SSI involving skin, subcutaneous tissue, and deeper soft tissues [23,24,25].

Patients were included if they had (1) undergone autologous microtia reconstruction, (2) developed a clinically diagnosed SSI involving the reconstructed auricle, and (3) received salvage-oriented management aimed at preserving the auricular framework. Patients were excluded if clinical records were incomplete, if follow-up duration was less than three months after initiation of infection management, or if immediate total framework removal was performed without a salvage attempt. A minimum follow-up period of three months was required to allow the completion of wound healing and early scar maturation and stabilization for outcome assessment. Ultimately, nine patients met the inclusion criteria and were analyzed.

### 2.2. Ethical Compliance

This study was conducted in accordance with the principles of the Declaration of Helsinki. The study protocol was reviewed and approved by the Institutional Review Board of Kangbuk Samsung Hospital (IRB No. 2025-12-020). Due to the retrospective design and use of anonymized clinical data, the requirement for informed consent was waived.

### 2.3. Infection Evaluation and Antibiotic Management

Upon clinical suspicion of SSI, wound cultures were obtained whenever feasible prior to initiation of targeted antimicrobial therapy. Empirical intravenous antibiotics were promptly initiated based on clinical severity and local antimicrobial resistance patterns and were subsequently adjusted according to culture and sensitivity results.

The timing of infection onset was classified as early or delayed. Early infection was defined as SSI occurring before complete postoperative wound healing was achieved, whereas delayed infection was defined as SSI developing after complete wound healing. The presence of auricular framework exposure, and degree of soft-tissue compromise, were documented as part of routine clinical assessment. Antibiotic therapy was continued until clinical resolution of infection, as indicated by normalization of local wound findings.

### 2.4. Negative-Pressure Wound Therapy

**Figure 1:**
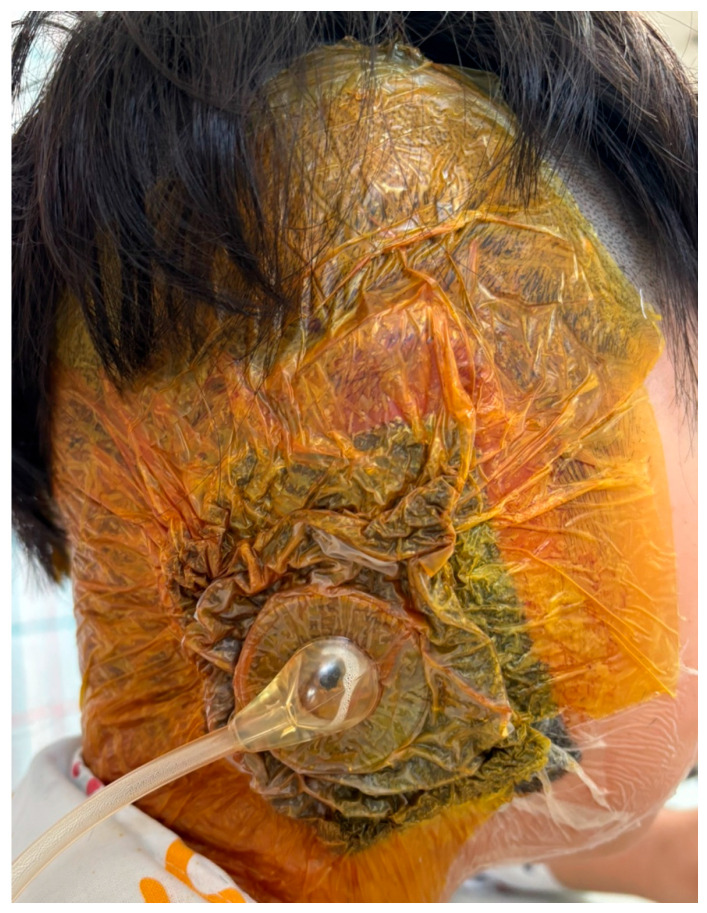
Application of negative-pressure wound therapy (NPWT) for surgical site infection following microtia reconstruction.

Negative-pressure wound therapy (NPWT) was selectively employed in patients presenting with wound dehiscence, persistent exudate, or exposed auricular framework. NPWT was applied using a commercially available system (CGbio Curasys, CGbio, Seoul, Republic of Korea), with continuous negative pressure maintained at −110 mmHg (Figure 1).

NPWT dressings were routinely changed every other day. The duration of NPWT varied among patients and was determined by clinical improvement, specifically resolution of infection-related signs and achievement of wound stabilization. Following initial infection control with systemic antibiotics, NPWT was used to stabilize the wound environment by reducing bacterial burden, promoting granulation tissue formation, and facilitating preservation of exposed cartilage [5,20,21,22]. NPWT was discontinued when infection control and wound stabilization were clinically achieved, rather than after a predetermined time interval. This approach aligns with prior reports demonstrating successful salvage of exposed microtia cartilage using NPWT as a temporizing modality rather than immediate framework removal [5,21,22].

### 2.5. Conservative Staged Debridement Strategy

After wound stabilization, management focused on infection control and maximal preservation of viable cartilage. Wound status was assessed daily during the acute phase of infection management. Conservative staged debridement was undertaken only after clear clinical demarcation of nonviable tissue was observed.

Nonviable tissue was defined clinically as dead, avascular tissue lacking bleeding or perfusion. Aggressive early debridement of cartilage was intentionally avoided, given the avascular nature of rib cartilage and the potential for secondary necrosis if premature excision was performed [5]. Debridement was performed only after clear demarcation of nonviable tissue was confirmed, following a standardized institutional approach. Instead, limited soft-tissue debridement combined with infection control and wound stabilization was prioritized, consistent with a salvage-oriented treatment philosophy [5,26].

### 2.6. Outcome Assessment and Follow-Up

Treatment outcomes were assessed based on the following endpoints: (1) resolution of infection without recurrence, (2) preservation of the auricular framework, and (3) achievement of stable wound healing. Follow-up duration was calculated from initiation of infection management to the most recent outpatient visit. Clinical outcomes were evaluated through physical examination and photographic documentation during follow-up visits.

## 3. Results

### 3.1. Patient Demographics

Table 1 summarizes patients’ data in the present study. During the study period, a total of 312 autologous microtia reconstructions were performed at our institution, including 176 rib cartilage framework implantations representing the first stage of two-staged auricular reconstruction; 136 auricular elevation procedures constituting the second stage; and 57 secondary touch-up procedures. Among these procedures, a subset of patients developed postoperative surgical site infection requiring active intervention. Based on predefined inclusion criteria, nine patients were identified and included in the final analysis. The cohort comprised four male and five female patients, none of whom had underlying medical comorbidities such as diabetes mellitus, immunologic disorders, or chronic systemic disease. Surgical site infection occurred at variable time points, ranging from the early postoperative period to delayed presentation following reconstruction. The mean follow-up duration was 9.7 months (range, 6–20 months).

### 3.2. Case Presentations

#### 3.2.1. Case 1—Early Postoperative Coagulase-Negative *Staphylococcus* Infection with Partial Framework Necrosis

**Figure 2:**
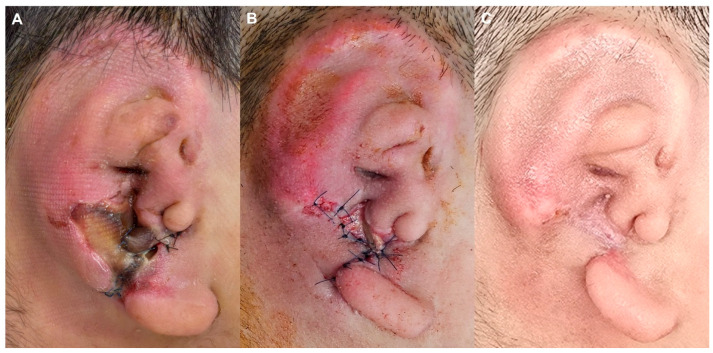
Early postoperative coagulase-negative *Staphylococcus* infection. (**A**) Postoperative day 10 showing purulent discharge and partial cartilage necrosis at the postauricular incision. (**B**) Early postoperative appearance after staged debridement and wound edge approximation. (**C**) Follow-up photograph demonstrating stable soft-tissue coverage and preservation of overall auricular contour despite limited tail cartilage loss.

A 10-year-old male developed surgical site infection on postoperative day 10 following rib cartilage-based auricular reconstruction. Clinical findings included purulent discharge from the postauricular incision and partial necrosis of the cartilage framework (Figure 2A). Empirical intravenous vancomycin was initiated, and wound cultures grew coagulase-negative *Staphylococcus*. Due to hypersensitivity to vancomycin, antimicrobial therapy was transitioned to intravenous teicoplanin. Local wound management included serial irrigation, negative-pressure wound therapy, and staged debridement. As tissue demarcation progressed, limited excision of nonviable cartilage was performed, followed by gradual wound closure. The infection resolved completely with preservation of the majority of the auricular framework. Approximately 2.5 × 2.0 cm of tail cartilage was lost; however, the overall auricular contour was maintained, with stable soft-tissue coverage at follow-up (Figure 2B,C).

#### 3.2.2. Case 2—Early Postoperative *Serratia marcescens* Infection

**Figure 3:**
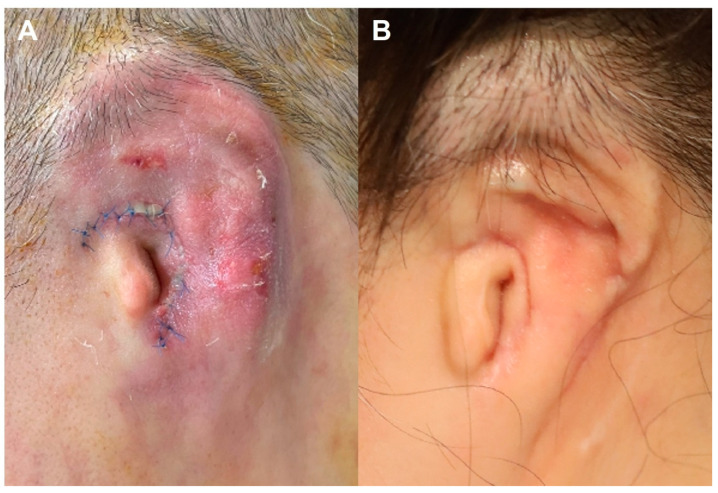
Early postoperative *Serratia marcescens* infection. (**A**) Postoperative day 7 demonstrating erythema and purulent discharge at the postauricular incision following rib cartilage framework implantation. (**B**) Post-treatment appearance showing resolution of infection and preservation of auricular contour after limited excision of nonviable helix-tail cartilage.

A 51-year-old female developed clinical signs of infection on postoperative day 7 following left rib cartilage framework implantation. Examination revealed erythema, tenderness, and purulent discharge from the postauricular incision site (Figure 3A). Initial incision and drainage were performed, and empirical intravenous cefazedone was initiated. Wound cultures subsequently identified *Serratia marcescens*, which demonstrated resistance to cefazolin and amoxicillin–clavulanate but susceptibility to piperacillin–tazobactam and ciprofloxacin. Antimicrobial therapy was escalated to piperacillin–tazobactam based on susceptibility results. Negative-pressure wound therapy (NPWT) was applied with dressing changes every other day to control exudate and promote granulation. Progressive demarcation of nonviable tissue allowed conservative excision of a small segment of helix-tail cartilage measuring approximately 1.0 × 0.5 cm, while the remaining framework remained viable. The infection resolved with preservation of the overall auricular contour, and the patient was discharged after clinical improvement (Figure 3B).

#### 3.2.3. Case 3—Late Traumatic *Pseudomonas aeruginosa* Infection

**Figure 4:**
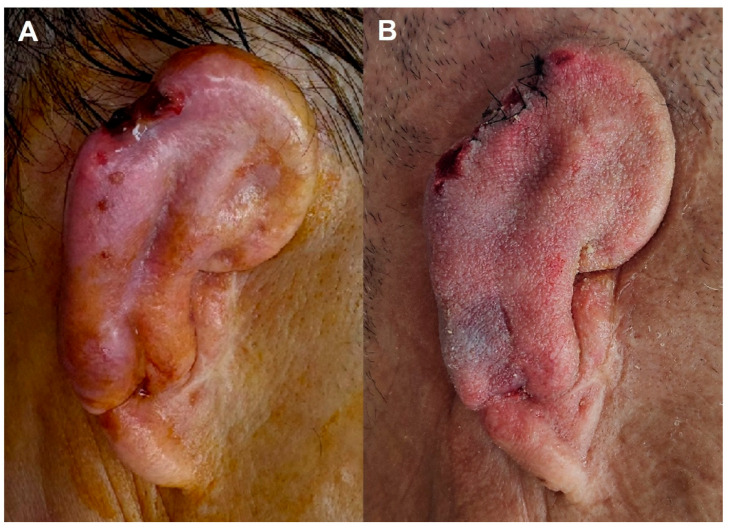
Late traumatic *Pseudomonas aeruginosa* infection. (**A**) Late presentation nearly 20 years after primary microtia reconstruction showing localized inflammation, necrotic eschar, and exposure of an internal support wire following minor trauma. (**B**) Intraoperative view after selective debridement of rigid, exposed cartilage enabling tension-free primary closure.

A 64-year-old male presented nearly 20 years after right-sided microtia reconstruction following minor trauma that resulted in exposure of an internal support wire. Clinical findings included localized erythema, swelling, necrotic eschar, and purulent discharge (Figure 4A). Initial debridement and primary closure were performed, and empirical intravenous vancomycin was administered. Wound cultures subsequently grew *Pseudomonas aeruginosa*, prompting a change in antimicrobial therapy to piperacillin–tazobactam. Adjunctive antibiotic solution irrigation and NPWT were applied to control local infection and manage exudate. Due to the increased rigidity of the ossified framework, in contrast to the pliable cartilage typically encountered in younger patients, primary closure over the exposed cartilage did not provide stable soft-tissue coverage. Consequently, selective debridement was performed to remove portions of exposed cartilage that imposed excessive tension on the approximated skin flap. This allowed for tension-free primary closure (Figure 4B). The absence of microcirculatory compromise facilitated effective bacterial eradication with systemic antibiotic therapy and enabled durable wound coverage without the need for additional reconstructive procedures. Follow-up wound cultures were negative, and the infection resolved without significant distortion of the long-standing reconstructed auricle.

#### 3.2.4. Case 4—Post-Elevation Multidrug-Resistant *Enterobacter* Infection

**Figure 5:**
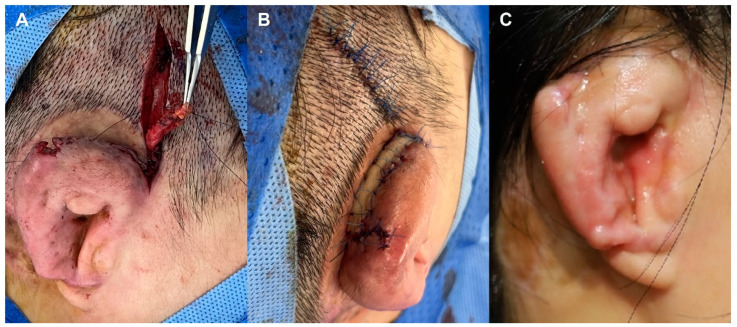
Post-elevation multidrug-resistant *Enterobacter* infection. (**A**) Coverage of exposed cartilage using a superficial temporal fascia flap after staged debridement. (**B**) Full-thickness skin graft applied over the fascia flap for definitive soft-tissue coverage. (**C**) Final postoperative appearance demonstrating stable wound healing with preservation of the upper two-thirds of the reconstructed auricle despite partial tail cartilage loss.

A 50-year-old female experienced a complicated postoperative course following staged microtia reconstruction. After initial rib cartilage grafting and subsequent ear elevation, partial skin graft necrosis and flap thinning were observed at the upper posterior helix region 2 months postoperatively. Despite initial debridement and primary approximation suture, the patient later developed wound dehiscence with cartilage exposure. Wound cultures identified *Enterobacter hormaechei*, followed by *Enterobacter cloacae* complex on repeat cultures. Both organisms exhibited multidrug resistance, including resistance to piperacillin–tazobactam and most cephalosporins, while remaining susceptible to carbapenems and fluoroquinolones. Antimicrobial therapy was escalated to ertapenem based on susceptibility testing. Prolonged NPWT was applied with regular dressing changes, promoting granulation over exposed cartilage and preventing further soft-tissue breakdown. Staged debridement allowed the selective removal of clearly nonviable cartilage only. Definitive soft-tissue reconstruction was achieved using a superficial temporal fascia flap (STFF) (Figure 5A) with full-thickness skin grafting (Figure 5B). Although approximately 2.0 × 1.5 cm of tail cartilage was lost, the primary framework and upper two-thirds of the auricular structure were successfully salvaged, with complete resolution of infection (Figure 5C).

### 3.3. Comparative Summary

Across all nine infections, early culture acquisition enabled a timely transition to targeted antimicrobial therapy, which was especially important in multidrug-resistant Gram-negative infections. NPWT played a consistently beneficial role in stabilizing wounds, reducing bacterial burden, and promoting granulation. Conservative staged debridement prevented the unnecessary removal of viable cartilage, and although the subject patients experienced limited cartilage loss, all retained overall auricular framework integrity. These shared outcomes support a structured salvage-oriented approach rather than aggressive early debridement.

## 4. Discussion

Surgical site infection (SSI) represents one of the most formidable complications in autologous microtia reconstruction, primarily due to the avascular nature of the rib cartilage framework and the limited reserve of the surrounding soft-tissue envelope [18,19]. Once infection develops, progressive soft-tissue inflammation, wound dehiscence, and cartilage exposure can rapidly destabilize the reconstruction, placing the entire framework at risk [14]. Historically, such scenarios frequently culminated in partial or total framework removal [18]. In contrast, the present case series demonstrates that a structured, salvage-oriented strategy can successfully preserve auricular architecture even in the context of severe or multidrug-resistant infection [5,21].

### 4.1. Microbiologic Considerations and Implications for Management

The spectrum of pathogens identified in this study—including *Serratia marcescens*, *Pseudomonas aeruginosa*, *Enterobacter cloacae*/*hormaechei* complex, and coagulase-negative *Staphylococci*—reflects the heterogeneity of organisms reported in microtia-related SSIs [14]. While Gram-positive organisms remain predominant in most large epidemiologic analyses, Gram-negative pathogens constitute a clinically meaningful subset and are often associated with antimicrobial resistance [26]. This heterogeneity underscores a critical principle: empiric antibiotic therapy alone is insufficient in many cases, and early culture acquisition with a prompt transition to targeted antimicrobial therapy is essential for infection control and framework preservation [26,27].

### 4.2. Antimicrobial Strategy as a Foundation for Salvage

In all cases, broad-spectrum empirical intravenous antibiotics were initiated immediately upon diagnosis, followed by culture-directed adjustment [25]. Notably, infections caused by *Enterobacter* species required escalation to carbapenem therapy, whereas *Pseudomonas* and *Serratia* infections were successfully managed with piperacillin/tazobactam-based regimens [25,27,28]. These findings mirror published resistance patterns in surgical infections and reinforce that antimicrobial management is not ancillary but foundational to any salvage attempt [27,28]. Effective infection control creates the necessary conditions for subsequent local wound management and tissue recovery [5].

### 4.3. Central Role of Negative-Pressure Wound Therapy

Negative-pressure wound therapy (NPWT) emerged as a cornerstone of salvage management across all patients. Beyond simple exudate control, NPWT provided a controlled wound environment that reduced the bioburden, stabilized exposed cartilage, and promoted granulation tissue formation [20,21,22]. Importantly, NPWT functioned as a *temporal bridge*, allowing sufficient time for antimicrobial therapy to take effect and for tissue viability to declare itself [26]. Prior reports on exposed microtia cartilage have emphasized that immediate framework removal is not obligatory, even in the presence of exposure or infection [5,21]. Instead, NPWT has been shown to facilitate successful salvage by preventing desiccation, minimizing bacterial proliferation, and enabling delayed definitive coverage [20,26]. The present findings reinforce these observations and support NPWT as a standard component of salvage-oriented SSI management rather than a rescue measure of last resort [15,20,21,22].

### 4.4. Conservative Staged Debridement over Radical Excision

A key insight from this series is the importance of conservative staged debridement. The avascular nature of rib cartilage makes early assessment of viability unreliable during active infection [18,19]. Aggressive early debridement risks unnecessary cartilage loss and may convert a salvageable framework into an irretrievable defect. In contrast, delaying cartilage excision until necrosis becomes clearly demarcated allows preservation of cartilage segments that often recover once infection is controlled [14,21]. This approach represents a paradigm shift from earlier dogma favoring radical excision and aligns with contemporary salvage strategies reported in the microtia reconstruction literature [18,19].

### 4.5. Soft-Tissue Envelope Restoration and Definitive Coverage

Soft-tissue compromise was a consistent precipitating factor for cartilage exposure in this cohort [19]. NPWT facilitated the gradual improvement of wound conditions, after which definitive coverage could be tailored to the extent of tissue loss [14,21]. Secondary intention healing was sufficient in select cases, whereas others required local flap advancement or superficial temporal fascia flap coverage with skin grafting. These experiences highlight that successful salvage depends not only on infection control but also on restoring a durable soft-tissue envelope capable of long-term framework protection [14,20,21].

### 4.6. Salvage as the Default Objective

Despite variability in the pathogen profile, infection timing, and degree of tissue compromise, all patients in this series achieved preservation of the auricular framework [14,21]. Although limited cartilage loss occurred in some cases, none required complete framework removal [14]. These outcomes support the principle that salvage should be considered the *default objective* in SSI following autologous microtia reconstruction [14,21,26]. Framework removal should be reserved for exceptional situations involving uncontrolled infection or complete structural devitalization [18,19].

### 4.7. Proposed Salvage-Oriented Management Protocol

Based on shared clinical patterns observed in this series and supported by the existing literature, the following structured protocol is proposed:

1. Early recognition of SSI with prompt clinical diagnosis.
2. Immediate wound culture acquisition and initiation of broad-spectrum intravenous antibiotics.
3. Rapid transition to culture-directed antimicrobial therapy.
4. Application of NPWT for wound stabilization, bioburden reduction, and cartilage protection.
5. Conservative staged debridement limited to clearly nonviable tissues.
6. Definitive soft-tissue coverage once infection has resolved and healthy granulation tissue is established.

**Figure 6:**
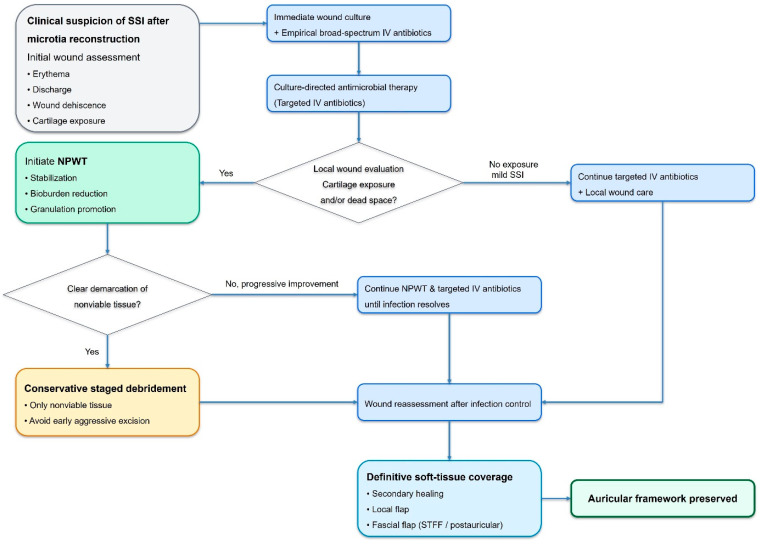
Salvage-oriented treatment algorithm for surgical site infection (SSI) after autologous microtia reconstruction. Upon clinical suspicion of surgical site infection, early wound culture acquisition and immediate initiation of empirical intravenous antibiotics are essential. Antimicrobial therapy is promptly tailored according to culture and sensitivity results. In cases with cartilage exposure, persistent drainage, or dead space, negative-pressure wound therapy (NPWT) is applied to stabilize the wound environment, reduce bioburden, and promote granulation tissue formation. Conservative staged debridement is performed only after clear demarcation of nonviable tissue. Definitive soft-tissue coverage is achieved once infection is controlled and healthy granulation is present. Framework removal is reserved for exceptional cases of uncontrolled infection or complete structural devitalization.

Figure 6 illustrates our salvage-oriented treatment algorithm for SSI in autologous microtia reconstruction. This algorithm emphasizes disciplined infection control, patience in tissue assessment, and prioritization of framework preservation.

### 4.8. Limitations

Several limitations warrant consideration. The retrospective design and small sample size limit generalizability, and all cases originated from a single institution, introducing potential treatment bias. In addition, the timing of SSI onset demonstrated marked heterogeneity, ranging from early postoperative infections to very late presentations occurring up to two decades after reconstruction, which may reflect distinct pathophysiologic mechanisms and further limits the uniform interpretation of outcomes.

Only a subset of patients underwent conservative staged debridement, reflecting both the limited cohort size and variability in clinical presentation; therefore, the relative contribution of staged debridement to salvage success cannot be definitively isolated. The absence of a control group precludes direct comparison with strategies involving early framework removal. Additionally, objective esthetic outcomes were not systematically quantified.

Accordingly, while the present findings suggest that a staged, salvage-oriented treatment strategy may be beneficial, they should be interpreted cautiously and not regarded as universally generalizable. Nonetheless, given the relative rarity of microtia-related SSI and the ethical constraints surrounding randomized intervention in such high-stakes complications, this series provides meaningful clinical insights and may serve as a foundation for future multi-center, prospective investigations.

## 5. Conclusions

Surgical site infection following autologous rib cartilage microtia reconstruction is a rare but potentially devastating complication, as infection of the avascular cartilage framework can rapidly compromise structural integrity [18,19]. This case series demonstrates that successful salvage is achievable without routine framework removal, even in severe infections, when a structured, salvage-oriented strategy is applied. Effective management consistently relied on early clinical recognition, prompt wound culture acquisition, and a timely transition to culture-directed antimicrobial therapy [25]. Negative-pressure wound therapy played a central role by stabilizing the wound environment, facilitating continuous drainage, reducing the bioburden, and promoting granulation tissue formation over exposed cartilage, thereby allowing time for infection control and tissue recovery [14,20,26]. Conservative staged debridement—performed only after clear demarcation of nonviable tissue—further enabled preservation of cartilage that might otherwise have been lost with aggressive early excision [14]. Definitive soft-tissue coverage, tailored to wound conditions, completed the salvage process.

In conclusion, surgical site infection after autologous microtia reconstruction can be effectively salvaged through a structured, timing-based algorithm emphasizing early culture-guided antimicrobial therapy, negative-pressure wound therapy, and conservative staged intervention, thereby maximizing framework preservation and optimizing clinical outcomes.

## Figures and Tables

**Table 1:** Summary of patients’ data.

Case	Age/Sex	Time of Wound Problem Occurrence	Wound Status at Presentation	Pathogen of Wound Culture	Gram Stain	MDR Pathogen	Treatment Modalities	Treatment Duration	Final Result	Follow-Up Periods
1	10/M	Postoperative 10 days after RCG	Skin flap erythema, purulent discharge	Coagulase-negative *Staphylococcus*	G(+) cocci	No	IV antibiotics, NPWT, staged debridement	18 days	Healed with loss of less than 10% cartilage framework	6 months
2	51/F	Postoperative 7 days after RCG	Skin flap erythema, swelling, purulent discharge	*Serratia marcescens*	G(−) rod	No	IV antibiotics, NPWT	7 days	Healed with no loss of cartilage framework	12 months
3	64/M	Postoperative 20 years after AE	Minimal cartilage exposure, purulent discharge, erythema	*Pseudomonas aeruginosa*	G(−) rod	Yes	IV antibiotics, NPWT, staged debridement	14 days	Healed with loss of less than 10% cartilage framework	6 months
4	50/F	Postoperative 2 months after AE	Skin graft partial necrosis, cartilage exposure	*Enterobacter hormaechei*, *Enterobacter cloacae*	G(−) rod	Yes	IV antibiotics, NPWT, staged debridement, STFF + FTSG	24 days	Lower two-thirds of framework preserved with upper helix loss	20 months
5	58/M	Postoperative 8 days after RCG	Skin flap erythema with purulent discharge	*Staphylococcus aureus*	G(+) cocci	No	IV antibiotics, NPWT	7 days	Healed without significant cartilage loss	9 months
6	52/F	Postoperative 14 days after RCG	Localized wound dehiscence with purulent discharge	Coagulase-negative *Staphylococcus*	G(+) cocci	No	IV antibiotics + NPWT	10 days	Healed without significant cartilage loss	10 months
7	48/F	Postoperative 11 days after RCG	Partial wound breakdown with minimal cartilage exposure	*Staphylococcus aureus*	G(+) cocci	No	IV antibiotics, NPWT	8 days	Healed without significant cartilage loss	15 months
8	58/M	Postoperative 12 days after RCG	Skin flap erythema with purulent discharge	*Enterobacter cloacae* complex	G(−) rod	No	IV antibiotics, NPWT	17 days	Healed without significant cartilage loss	8 months
9	54/F	Postoperative 2 months after RCG	Delayed wound breakdown with purulent discharge	*Pseudomonas aeruginosa*	G(−) rod	No	IV antibiotics, NPWT	21 days	Healed without significant cartilage loss	12 months

M, male; F, female; RCG, rib cartilage graft; AE, auricular elevation; G(+), Gram-positive; G(−), Gram-negative; IV, intravenous; NPWT, negative-pressure wound therapy; STFF, superficial temporalis fascia flap; FTSG, full-thickness skin graft.

## Data Availability

The original contributions presented in this study are included in the article. Further inquiries can be directed to the corresponding author.
